# Metabolomic analysis reveals potential biomarkers and the underlying pathogenesis involved in *Mycoplasma pneumoniae* pneumonia

**DOI:** 10.1080/22221751.2022.2036582

**Published:** 2022-02-21

**Authors:** Jieqiong Li, Laurence Don Wai Luu, Xiaoxia Wang, XiaoDai Cui, Xiaolan Huang, Jin Fu, Xiong Zhu, Zhenjun Li, Yi Wang, Jun Tai

**Affiliations:** a Department of Respiratory Disease, Beijing Pediatric Research Institute, Beijing Children’s Hospital, Capital Medical University, National Center for Children’s Health, Beijing, People’s Republic of China; b School of Biotechnology and Biomolecular Sciences, University of New South Wales, Sydney, Australia; c Central & Clinical Laboratory of Sanya People’s Hospital, Sanya, People’s Republic of China; d Experimental Research Center, Capital Institute of Pediatrics, Beijing, People’s Republic of China; e State Key Laboratory for Infectious Disease Prevention and Control, National Institute for Communicable Disease Control and Prevention, Chinese Center for Disease Control and Prevention, Beijing, People’s Republic of China; f Department of Otolaryngology, Head and Neck Surgery, Children's Hospital Capital Institute of Pediatrics, Beijing, People’s Republic of China

**Keywords:** *Mycoplasma pneumoniae* pneumonia*;* metabolomics, diagnosis, pathogenesis, children

## Abstract

Although previous studies have reported the use of metabolomics for infectious diseases, little is known about the potential function of plasma metabolites in children infected with *Mycoplasma pneumoniae* (MP). Here, a combination of liquid chromatography-quadrupole time-of-flight mass spectrometry and random forest-based classification model was used to provide a broader range of applications in MP diagnosis. In the training cohort, plasma from 63 MP pneumonia children (MPPs), 37 healthy controls (HC) and 29 infectious disease controls (IDC) was collected. After multivariate analyses, 357 metabolites were identified to be differentially expressed among MPP, HC and IDC groups, and 3 metabolites (568.5661, 459.3493 and 411.3208) had high diagnostic values. In an independent cohort with 57 blinded subjects, samples were successfully classified into different groups, demonstrating the reliability of these biomarkers for distinguishing MPPs from controls. A metabolomic signature analysis identified major classes of glycerophospholipids, sphingolipids and fatty acyls were increased in MPPs. These markedly altered metabolites are mainly involved in glycerophospholipid and sphingolipid metabolism. As the ubiquitous building blocks of eukaryotic cell membranes, dysregulated lipid metabolism indicates damage of the cellular membrane and the activation of immunity in MPPs. Moreover, lipid metabolites, differentially expressed between severe and mild MPPs, were correlated with the markers of extrapulmonary complications, suggesting that they may be involved in MPP disease severity. These findings may offer new insights into biomarker selection and the pathogenesis of MPP in children.

## Introduction

*Mycoplasma pneumoniae* (MP) is an atypical pathogen and one of the most common causes of community-acquired pneumonia (CAP) [1, 2]. Although MP pneumonia (MPP) was once considered a “benign” infection, it is associated with a high percentage of morbidity and mortality. Severe forms of MP infection have heterogeneous clinical presentations including diffuse alveolar hemorrhage, cavitary lesions and acute respiratory disease syndrome (ARDS) [3–5]. Existing diagnostic techniques have several limitations for diagnosing pediatric MPP. For example, culture and serological tests are not appropriate for rapid detection because they are insensitive and time-consuming [6], and use of the polymerase chain reaction (PCR) method is also limited by the high cost and complicated instrumentation [7, 8]. Therefore, rapid and accurate diagnostic methods are required.

In general, detecting biomarkers in the plasma is a useful auxiliary method to disease diagnosis [9, 10]. Recently, advances in metabolomic approaches have promoted the comprehensive and unbiased evaluation of billions of circulating metabolites that are associated with bioactivity, regulation and dysregulation. Metabolomics has been extensively used in many infectious and noninfectious diseases [11–18]. Preliminary metabolomic studies have shown its suitability for the identification of novel discriminative metabolites with potential application in the diagnosis of early Alzheimer's disease [18]; early detection of type 2 diabetes [14]; diagnosis of active tuberculosis in HIV patients [12] and the diagnosis and monitoring of multiple sclerosis disease progression [17]. However, specific metabolites that can be used to discriminate MPPs from healthy controls (HCs) and other infectious disease controls (IDCs), especially in children, have not been identified.

For MP infection, the most severe damage occurs in the respiratory tract and lungs [1], and consequently, may alter the metabolites involved in inflammation. Changes in the metabolome are the ultimate response of a biological system to stimuli, and so, at least theoretically, these changes are a closer representation of the phenotype. This allows the assessment of cellular states and processes at a functional level [19]. Thus these metabolic disorders may indirectly reflect pulmonary inflammation and lead to multiple severe extrapulmonary complications. Additionally, some pathogens can evade the host's immune system by using host-derived lipid membranes during intercellular transmission. This allows them to expand and replicate unrestricted in the early stages of infection [20]. Failure to limit the infection at the initial stage can exacerbate disease symptoms and further contribute to severe pneumonia. The metabolome is highly dynamic, reflecting continuous changes of both metabolic and signaling pathways, and is sensitive to diverse host changes induced by infection [19]. Therefore, it is important to investigate whether host-derived metabolites in circulation are implicated in the pathogenesis of MP infection.

In this study, we combined liquid chromatography-quadrupole time-of-flight mass spectrometry and a random forest-based classification model to select the most discriminant markers from 63 MPPs, 37 HCs and 29 IDCs. The biomarkers were further validated on an independent cohort with 57 blinded subjects. Further investigations into altered metabolites revealed a significant impact of MP on lipid metabolism, especially pathways involving glycerophospholipid and sphingolipid metabolism that likely contribute to the pathogenesis of MPP. Differentially expressed metabolites associated with MPP disease severity were also analysed. These findings provide valuable knowledge about plasma biomarkers associated with MPP and an insight into the pathogenesis of MP infection.

## Material and methods

### Patients and controls

Patients and controls were recruited from Beijing Children’s Hospital from April 2016 to August 2018. Diagnosis of pediatric MPP was performed by the Chinese Medical Association guidelines as follows: (1) fever, acute respiratory symptoms (cough, tachypnea, difficulty breathing) or both; (2) low breathing or dry, wet rales; (3) chest film findings characterized by lung portal lymph node and lung gate shadow, bronchopneumonia, interstitial pneumonia, and large and high-density shadow; and (4) children with positive PCR results or MP antibody titer seroconversion from negative (<1:80) to positive (≥1:160) titers. Severe MPP was defined as MPP with one of the following: (1) poor general condition; (2) increased breathing rate; (3) cyanosis and dyspnea; (4) infiltration with multilobed or ≥2/3 of the lung; (4) transcutaneous oxygen saturation ≤92% in room air; (6) extrapulmonary complications [21]. IDC patients were enrolled by the standard that was published previously by our lab [22]. HC group subjects were enrolled from children who underwent a health checkup at Beijing Children’s Hospital. Patients with immunodeficiency or those taking immunosuppressants were excluded.

This research was approved by the Ethics Committee of Beijing Children’s Hospital. All the methods and research protocol in this research were conducted by the Ethics Committee’s existing guidelines.

### Evaluation of clinical characteristics and multiple markers

Clinical information was retrospectively collected from the medical records of patients. Complete information about the clinical symptoms and multiple systemic markers were reviewed. The patient demographics, clinical symptoms, inflammatory markers [C-reactive protein (CRP), white blood cell (WBC), procalcitonin (PCT), neutrophil% (NEUT%), lymphocyte% (LYMPH%)], multiple systemic indicators including digestive markers [aspartate aminotransferase (AST), alanine aminotransferase (ALT), total bile acids (TBA), γ-glutamyl transpeptidase (GGT), albumin (ALB), total bilirubin (TBIL), direct bilirubin (DBIL), indirect bilirubin (IBIL), alkaline phosphatase (ALP)], cardiovascular markers [hydroxybutyrate dehydrogenase (HBDB), lactate dehydrogenase (LDH), creatine kinase (CK), phosphocreatine kinase isoenzyme (CK-MB)], hematological markers [red blood cell (RBC), D-Dimer, fibrinogen (FIB), total protein (PT), platelet (PLT), AT-III, hemoglobin (Hgb)] and urinary markers [Urea, urinary creatinine (Cr)] were analysed and compared between severe and mild MPPs on admission.

### Liquid chromatography–mass spectrometry

A mixture of acetonitrile/methanol (75:25 v/v, 300 μL) was added to the plasma (100 μL) for protein deposition. After vortexing for 60 s, the mixture was left to rest for 10 min and centrifuged at 12,000 rpm for 10 min at 4°C. Syringe filters (0.22 mm, Jinteng) were used to filter the supernatant prior to LC/MS/MS analysis [23].

The Nexera X2 system (Shimadzu, Japan) and TripleTOF 5600 quadrupole-time-of-flight mass spectrometers (AB SCIEX, USA) were used for ultra-performance liquid chromatography combined with quadrupole time-of-flight tandem mass spectrometry (UPLC Q-TOF MS).

Liquid chromatography separation was performed on a ZORBAX Eclipse Plus C18 column (2.1 × 100, 3.5 mm, Agilent, USA) maintained at 45°C. The injected sample volume was 10 µL for each run in the positive and negative injection mode, and the flow rate of the mobile phase was 0.5 mL/min. The quadrupole analyser ranged from 50 to 1500 *m/z*. Independent reference lock-mass ions via Analyst TF 1.6 and MarkerView 1.2.1 (absciex, USA) were used to ensure mass accuracy during data acquisition. A peak table with retention time, m/z, and corresponding peak intensity was generated using MSDIAL ver3.70. The assigned modified metabolite ions were identified by database searches in the Human Metabolome Database (HMDB, http://www.hmdb.ca/spectra/ms/search).

### Statistical analysis

#### Statistical analysis of clinical data

Clinical indices were analysed using SPSS 16.0. Continuous data were analysed using a Student’s *t*-test and categorical data were tested using Fisher’s exact test. Data were expressed as mean ± SD. The level of significance was set at *P* < 0.05. Correlation analysis was performed using R version 3.6.1.

#### Statistical analysis of metabolomics

Missing values were imputed with the minimal value for each feature. Fold-change (FC) was calculated on the mean of the same patient group for each pair of comparing groups. Two-sided unpaired Welch’s *t*-test was performed for each pair of comparing groups. The statistical significant altered metabolites were selected using the following criteria: VIP > 1, *P* value < 0.05 and |FC| >1.5.

The Mann–Whitney *U* test was used to compare the MPP group with the HC or IDC group. Principal component analysis (PCA) was applied to visualize the distributions of the different groups on the mean-centered and Pareto-scaled data. Partial least squares-discriminate analysis (PLS-DA) was conducted using MetaboAnalyst 4.0 (http://www.metaboanalyst.ca/MetaboAnalyst/). The PLS-DA models were cross-validated using a 10-fold method with unit variance scaling.

Volcano plots were calculated using a combination of FC and *t* tests, and the intensity data of these regions were used in GraphPad analysis, hierarchical cluster analysis and metabolic pathway analysis. Heat maps of differential metabolites and relationships were displayed using the Multi Experiment Viewer software (MeV, version 4.7.4). Pathway analysis and visualization were applied using the Metaboanalyst4.0 web portal (http://www.metaboanalyst.ca/).

Connected networks of the differentially expressed metabolites were built and analysed in Metscape, which is a plug-in for Cytoscape (v.3.2.1). Metscape was used to build the network of metabolites, analyse the correlation of these different metabolites and visualize the compound networks.

### Selection of biomarker candidates

We used random forest analysis to select the top metabolites that were differentially expressed between the three groups. Then, we used receiver operating characteristic (ROC) curves to evaluate the accuracy of the metabolites in the validation sets. The diagnostic parameters, including sensitivity and specificity, were defined by the minimum distance to the top-left corner [24].

## Results

### Study design and patients

**Figure 1.**
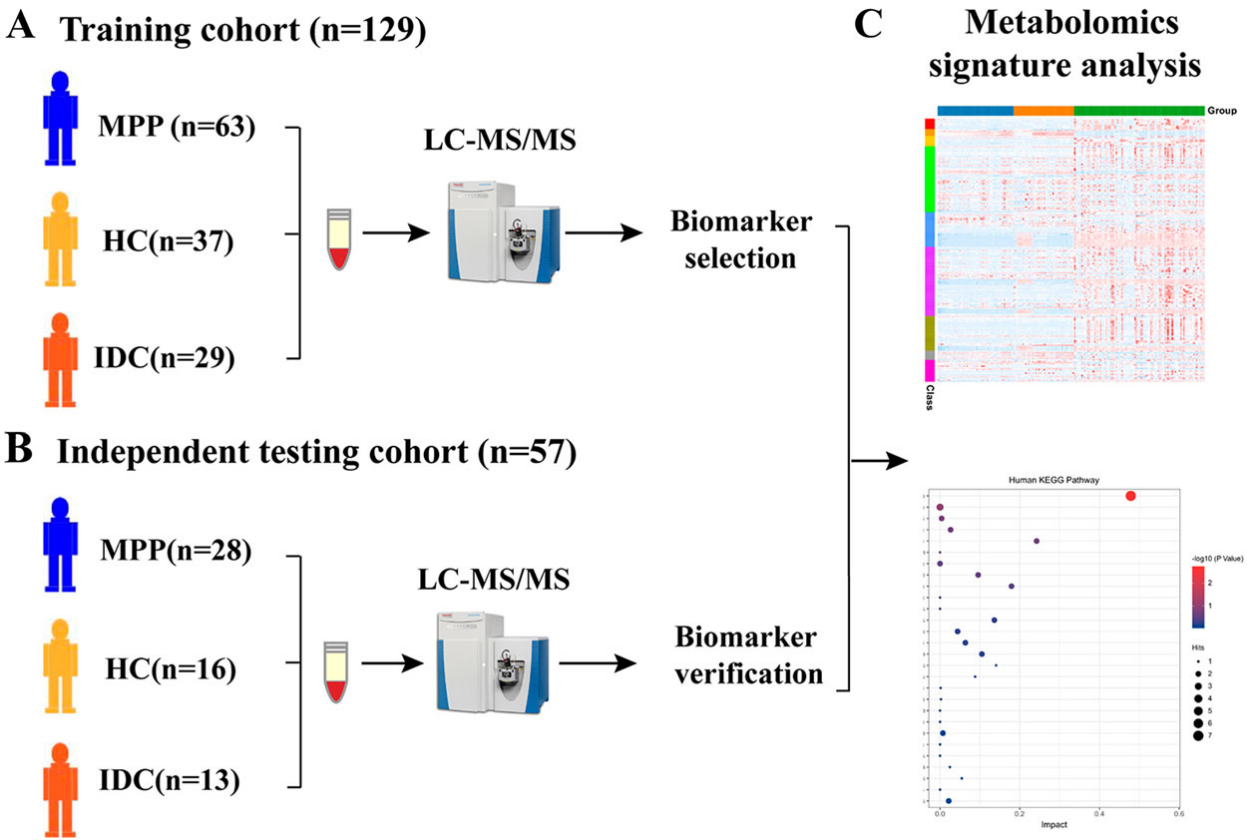
Study design and patients: (A) samples in a training cohort for metabolomic analysis; (B) verification of biomarkers in an independent testing cohort; (C) data from training and testing cohorts for metabolomics signature analysis.

The patients’ demographic characteristics are shown in Table 1. Sixty-three MPP patients, 37 HC children and 29 IDC patients were included in the training cohort for biomarker selection (Figure 1A) and 57 children, including 28 MPPs, 16 HCs and 13 IDCs were included in the independent testing cohort to verify the biomarkers (Figure 1B). The causative agents of IDC patients are shown in Table S1. All HCs and IDCs showed negative PCR results for MP.

**Table 1.** Demographic characteristics of participants enrolled.

	Training cohort (*n* = 129)				Independent testing cohort (*n* = 57)			
	MPP	HC	IDC	*P* value^a^	MPP	HC	IDC	*P* value^a^
Sample size	63	37	29	/	28	16	13	/
Gender(male/female)	36/27	22/15	16/13	0.438	15/13	9/7	8/5	0.512
Age(years)^b^	6.9 ± 3.0	6.3 ± 3.5	5.6 ± 3.9	0.323	7.0 ± 3.0	7.0 ± 3.0	6.2 ± 5.6	0.082
Range (years)	3–15	0.5–14	0.3–16	/	1–14	0.5–15	1–16	/

MPP, mycoplasma pneumoniae pneumonia; HC, healthy control; IDC, infectious disease control.

^a^ *P-*value among MPP, HC and IDC.

^b^ Data are presented as the mean ± SD.

The data from both training and testing cohorts were then combined to identify the characteristic metabolite changes of MPPs and provide an insight into the pathogenesis of MP infection. Moreover, differentially expressed metabolites between severe and mild MPPs, and their relationship with clinical indices were also analysed (Figure 1C).

### Biomarkers selection of MPP

We investigated the possibility of differentiating MPPs from HCs and IDCs based on the molecular metabolite signatures. After peak alignment and removal of missing values, 4523 positive-mode features were detected. To ensure reliability, a PCA score plot was generated (Figure S1A), which included control group, model group and QC samples. QC samples (blue) clustered together tightly, reflecting the stability of the instrument and showing that the quality of all the LC–MS data for this study was satisfactory. In addition, during the entire experiment, 90%, 47% and 12% of the metabolite RSD in the QC samples were within 30%, 15% and 5%, respectively (Figure S1B). These results illustrate the reliability of the analytical method used in this work.

**Figure 2.**
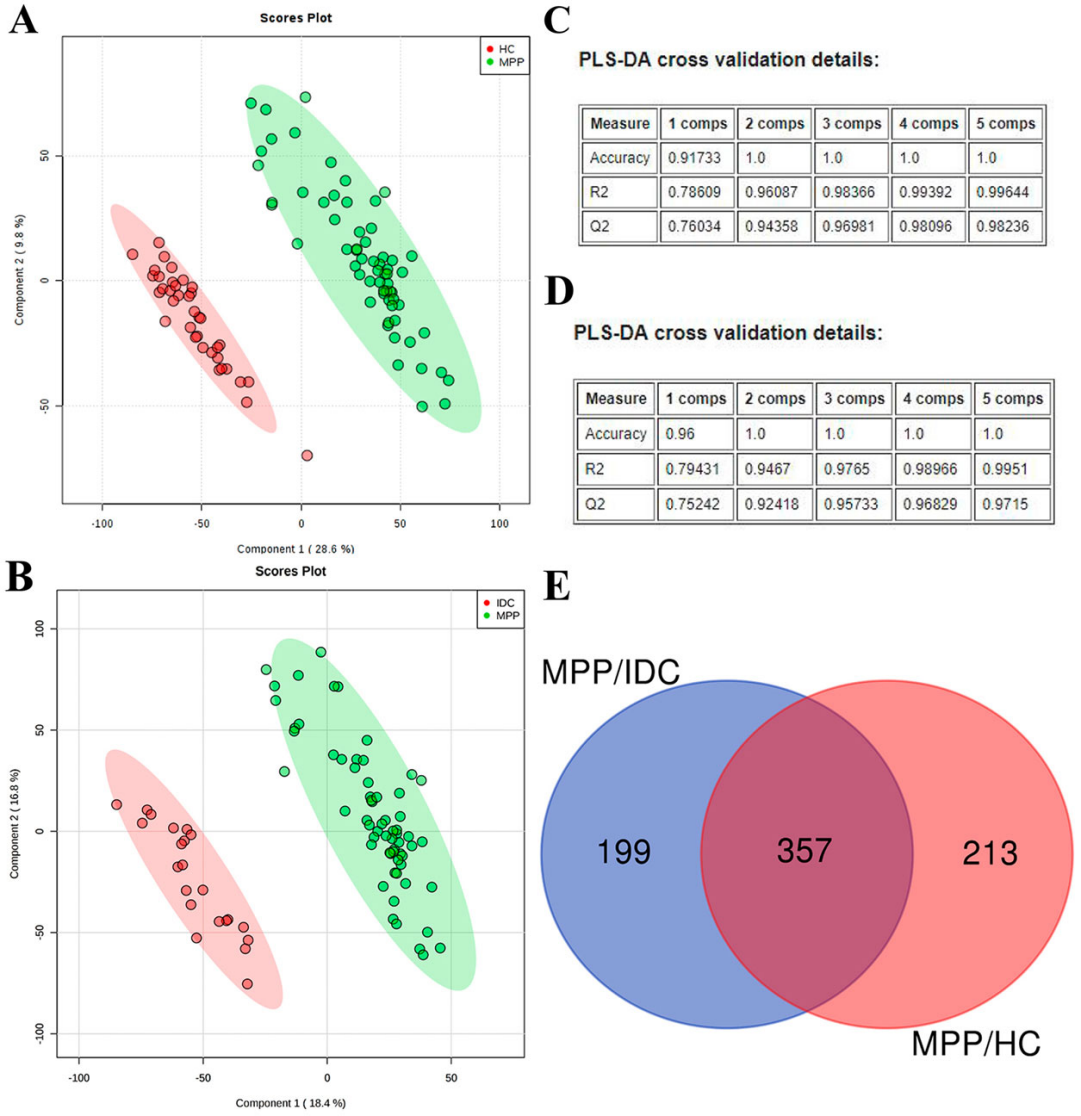
Identification of differentially expressed metabolites in MPPs: (A) PLS-DA score plots for the MPPs and HCs; (B) PLS-DA score plots for the MPPs and IDCs; (C) parameters for assessing the quality of the PLS-DA model for the MPPs and HCs; (D) parameters for assessing the quality of the PLS-DA model for the MPPs and IDCs; (E) differentially expressed metabolites identified in MPP children compared with IDC and HC.

Variables with VI*P* value >1.0, *P* value <0.05, and |FC| >1.5 were considered to be potential differential metabolites. A PLS-DA model was applied to characterize disturbances of MPPs with HCs (Figure 2A) and IDCs (Figure 2B). The model for classification of these groups obtained satisfactory validation, with *R*^2^ (cum)    0.996 and *Q*^2^ (cum)    0.982 between the MPP and HC groups (Figure 2C) and with *R*^2^ (cum)    0.995 and *Q*^2^ (cum) 0.972 between the MPP and IDC groups (Figure 2D).

**Figure 3.**
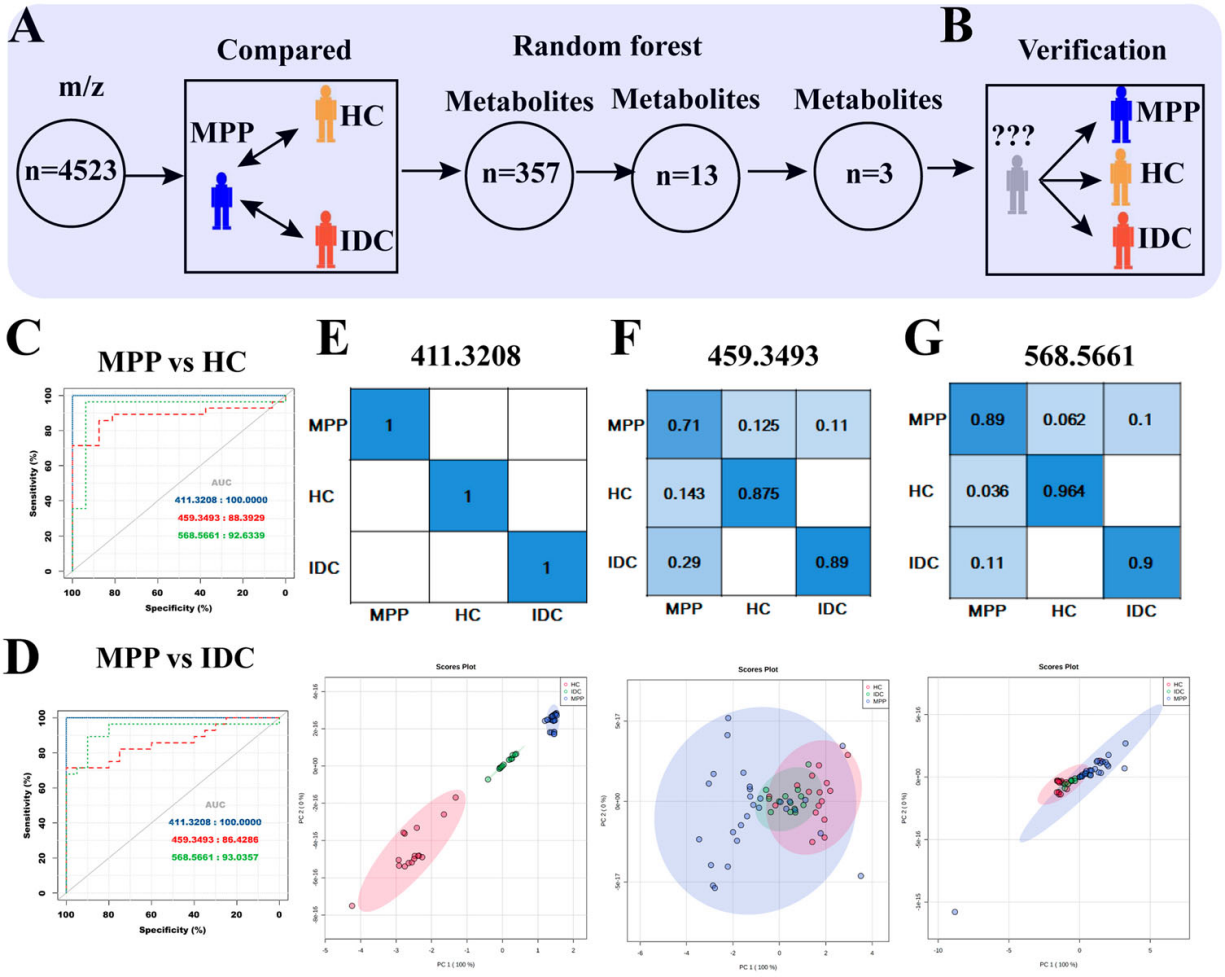
Identification and verification of potential biomarker combinations for the classification of MPP Patients: (A) the workflow for biomarker selection; (B) verification of biomarkers in an independent cohort with 57 blinded subjects; (C) AUC values of three biomarkers were calculated for the classification of MPPs and HCs; (D) AUC values of three biomarkers were calculated for the classification of MPPs and IDCs; (E) the confusion matrix and PCA analysis of 411.3208 among different plasma samples from cohort 2; (F) the confusion matrix and PCA analysis of 459.3493 among different plasma samples from cohort 2; (G) the confusion matrix and PCA analysis of 568.5661 among different plasma samples from cohort 2.

The analysis results showed that 570 and 556 metabolites differed between the MPPs and HCs or IDCs, respectively. Of these, 357 were overlapping (Figure 2E). A random forest analysis was performed to estimate the importance of each metabolite. As presented in Table S2, 13 metabolites had higher significance than the other metabolites, of which, 3 metabolites (411.3208, 568.5661 and 459.3493) showed high diagnostic values (Figure 3A and Table S3).

### Independent validation of biomarkers

To evaluate the reliability of the biomarkers, a randomized and blinded set was used for validation (Figure 3B). The AUC, sensitivity and specificity of the ROC curves generated from the verification set are presented in Figure 3(C–G) and Table S3. As shown in Figure 3(C), 411.3208, 459.3493, and 568.5661 had AUC values of 1, 0.884, 0.926 when MPP patients were compared with HCs, respectively. Additionally, the comparison of MPPs and IDCs also showed relatively high AUCs, ranging 0.864–1 (Figure 3D). Importantly, the metabolite 411.3208 (4a-formyl-5a-cholesta-8,24-dien-3b-ol) was able to completely distinguish MPP from HC or IDC in the validation set, with 100% sensitivity and specificity (Figure 3E and Table S3). For the other two metabolites, 459.3493 had a sensitivity of 85.7% and 71.4% and a specificity of 87.5% and 89%, respectively, for MPPs diagnosis compared with HCs or IDCs (Figure 3F and Table S3). 568.5661 (Cer (d18:0/18:0)) had a sensitivity of 93.8% and 89.3% and a specificity of 96.4% and 90.0%, respectively, for MPPs diagnosis compared with HCs or IDCs (Figure 3G and Table S3). The PCA showed that the samples were classified into different groups, indicating the reliability of these biomarkers for distinguishing MPP from controls (Figure 3E–G).

### Dysregulated lipid metabolism indicates membrane fusion damage and immune damage induced by MP infection

Pathogens are known to cause significant changes to the host cell metabolites by altering key pathways during infection [25]. Thus, to gain an insight into the pathogenesis of MPP, the data from the training and testing cohorts were combined to analyse the metabolomic signatures of MP infection.

#### Metabolite profiles of MPPs

**Figure 4.**
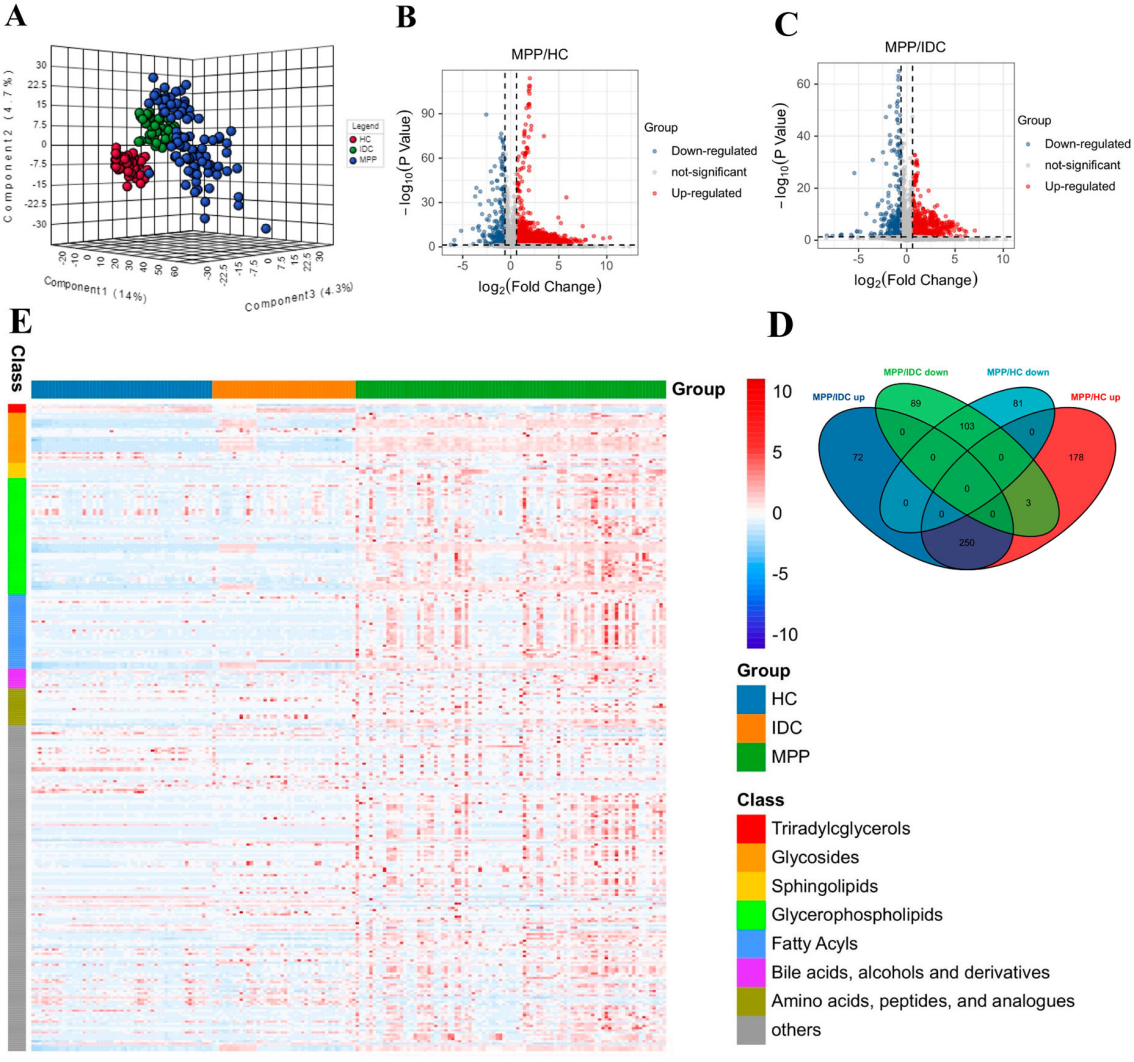
Analysis of the metabolomic signatures from patients with MPP. (A) The serum metabolic phenotypes of MPP-positive patients substantially differed from controls using PLS-DA. (B) The volcano plot derived from a targeted metabolomic analysis illustrates the top serum metabolites that were increased (shown in red) or decreased (shown in blue) in MPP-positive patients as compared with HCs. (C) The volcano plot derived from a targeted metabolomic analysis highlights the top serum metabolites that were increased (shown in blue) or decreased (shown in red) in MPP-positive patients as compared with IDCs. (D) Venn diagram displays the number of differentially expressed metabolites in MPPs compared to HCs and IDCs (|FC| >1.5, *P* < 0.05, VIP > 1). (E) Hierarchical clustering analysis revealed a significant impact of MPP on levels of triadylcglycerols, sphingolipids, glycerolipids, glycerophospholipids, fatty acyls, bile acids/alcohols/ derivatives and amino acids/peptides/ analogues. A vectorial version of this figure is provided in Table S3.

In the PLS-DA analysis, the MPP samples were separated from HCs and IDCs, illustrating evident differences in their plasma metabolite profiles (Figure 4A). Volcano plots from untargeted metabolomic analyses highlight the different metabolites that increased (red) or decreased (blue) in the plasma of MPPs, as compared to HCs and IDCs, respectively (Figure 4B and C). The overlapping differentially expressed metabolites between MPPs and HCs or IDCs were then selected for pathogenesis analysis (Figure 4D). Interestingly, we found that most differentially expressed metabolites in MPPs were concentrated in lipids, including glycerophospholipids, sphingolipids, triadylcglycerols and fatty acyls (Figure 4E); a detailed version of this figure is provided in Table S4. This result indicates that MP infection mainly affected lipid metabolism, which might play an important role in the pathogenesis of MPP.

#### Glycerophospholipid and sphingolipid metabolism is involved in MPP-related pathways

**Figure 5.**
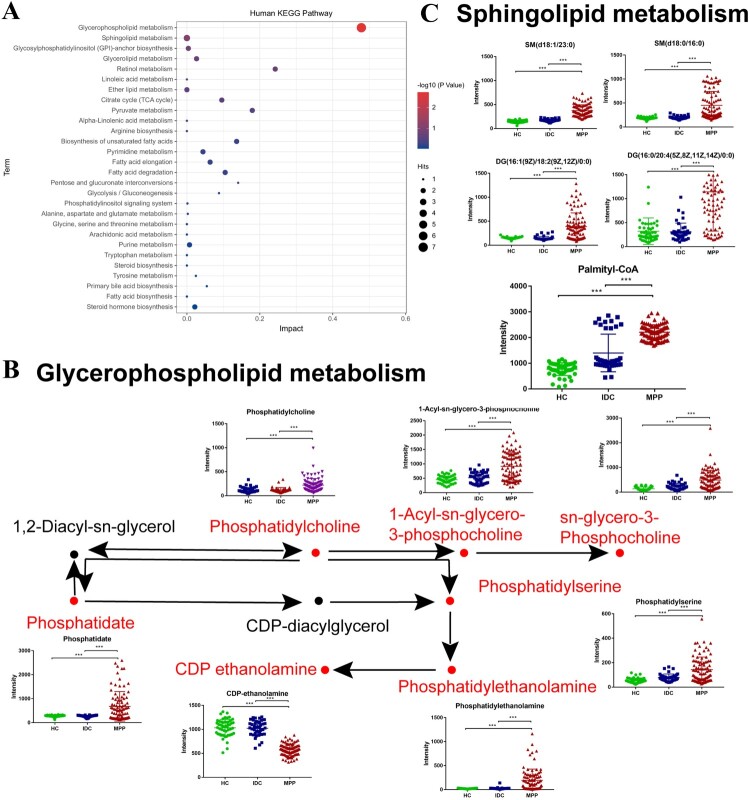
Pathway analysis of differentially expressed metabolites. (A) Impact factors of pathways calculated using the KEGG Pathway Database. (B) Significant changes were seen in the levels of some intermediates of the glycerophospholipid metabolism pathways in plasma of MPP samples. (C) Significant changes were seen in the levels of some intermediates of the sphingolipid metabolism pathways in plasma of MPP samples.

To obtain further metabolic pathway information about the different metabolites, the KEGG Pathway Database was used to analyse the above differentially expressed metabolites. Based on *P* value that were <0.05, metabolomics data revealed a significant impact of MPP on lipid metabolism, especially pathways in glycerophospholipid metabolism and sphingolipid metabolism (Figure 5A and Table S5).

The significant difference of plasma metabolites can reflect the metabolic changes and pathogenesis of MP infection [1]. Here, major classes of plasma glycerophospholipids including lysophosphatidic acid (LPA), lysophosphatidylcholine (LPC), phosphatidic acid (PA), phosphatidylcholine (PC), glycerophosphoserines (PS) and phosphatidylethanolamines (PE) were significantly increased in MPPs compared to HCs and IDCs (Figure S2). Most sphingolipids and fatty acyls were also significantly increased in MPPs compared to controls (Figure S3). Moreover, metabolites associated with glycerophospholids and sphignolipids metabolism, such as phosphatidate, 1-Acyl-sn-glycero-3-phosphocholine, palmityl-CoA, and diacylglycerol (DG) were also increased in MPPs (Figure 5B and C). As ubiquitous building blocks of eukaryotic cell membranes, a large body of evidence has demonstrated that sphingolipid metabolites are signaling molecules that regulate a diverse range of cellular processes that are important in immunity, inflammation and inflammatory disorders [26]. Glycerophospholids not only comprise the bulk of the cellular membrane bilayers but also regulate a variety of biological processes [27, 28]. Collectively, the high expression of glycerophospholids and sphingolipids in MPPs suggests both membrane fusion damage and dysregulated immunity induced by MP infection, consistent with previous studies [29].

#### Intricate correlation networks identify pathologically relevant metabolites

**Figure 6.**
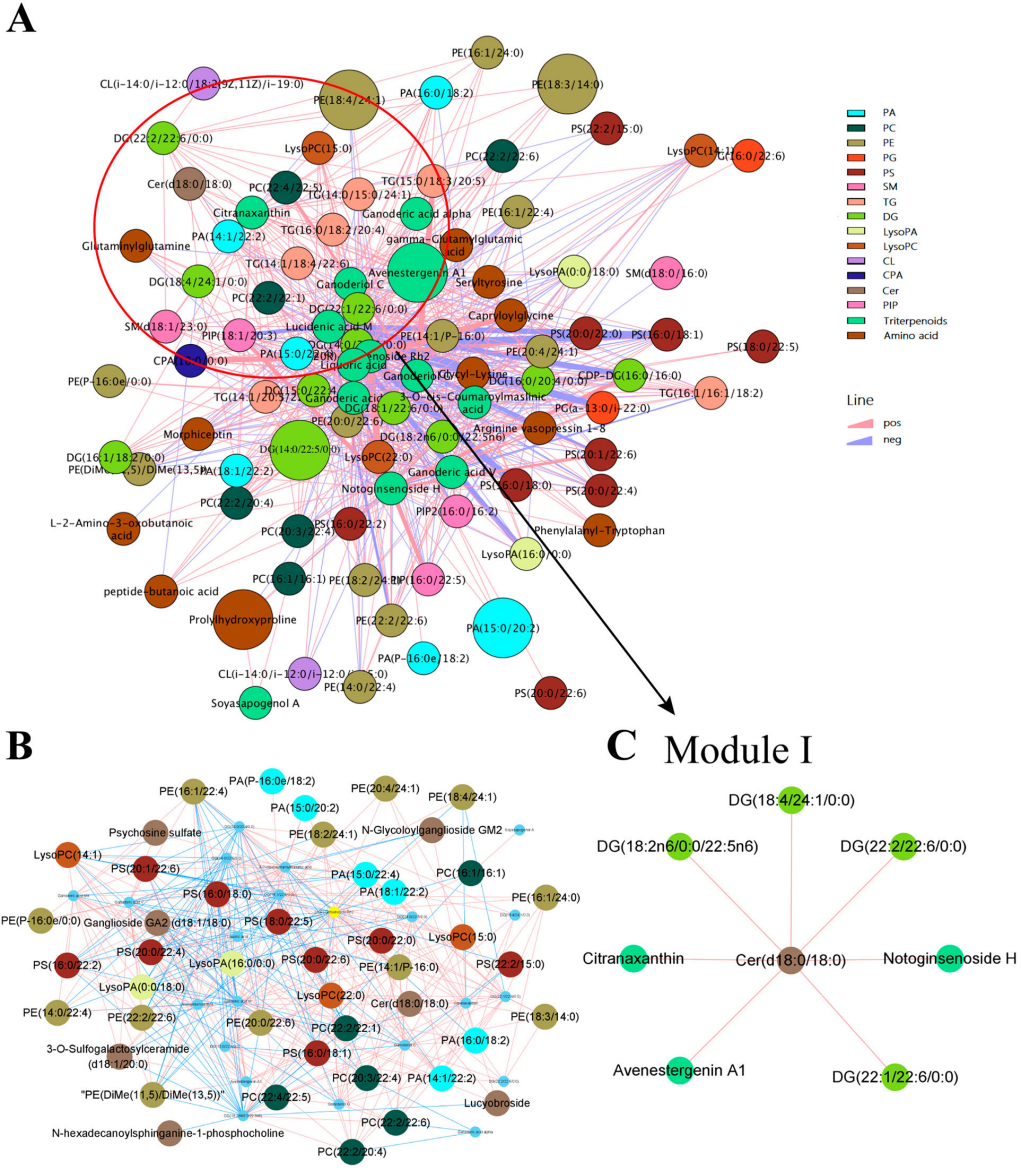
Multiscale embedded correlation network analysis illustrates the differential correlation of metabolites in MPPs relative to controls. (A) Only lipid and amino acids pairs with significant differential correlations (empirical *P* < 0.05) were included. Negative correlations are shown in purple and positive correlations are shown in pink. For important pathologically related metabolites, two modules of biological interest were circled and expanded for better visual clarity. (B) Glycerophospholipids and glycerolipids pairs with significant differential correlations (empirical *P* < 0.05) were included. These two classes of lipids created a complex network, which may play an essential role in MP infection. (C) Module I comprises of Cer (d18:0/18:0), as the hub, connected to DG (18:4/24:1/0:0), DG (22:2/22:6/0:0), DG (22:1/22:6/0:0) and DG (18:2n6/0:0/22:5n6).

To obtain additional insights about the relationship between metabolites, the different metabolites were selected to build a potential metabolic network using Cytoscape, a tool for interactive exploration using human metabolic networks (Figure 6A). Only differential lipids and amino acid correlations with empirical *P* < 0.05 were displayed. Strong correlations between metabolite levels can imply that these metabolites lie along a common metabolic pathway and are co-regulated, and that changing correlation patterns between metabolite-pairs in disease compared to healthy states can potentially indicate pathologically relevant metabolic dysregulation (Figure 6A). We noticed that most metabolites that contained glycerophospholipids, and glycerolipids were up-regulated in the MP groups and had highly correlated coefficients (Figure 6B).

Module I comprised of Cer (d18:0/18:0) as the hub connected to numerous DGs, [i.e. DG (18:4/24:1/0:0), DG (22:2/22:6/0:0), DG (22:1/22:6/0:0) and DG (18:2n6/0:0/22:5n6)] by pink lines, indicating a positive association between Cer (d18:0/18:0) and DG in MPPs. Cer (d18:0/18:0) belongs to the class of glycerolipid and the interrelationships of these lipids might reveal the direction of future potential mechanisms (Figure 6C). Collectively, our results suggest that glycerophospholipids, glycerolipids, and metabolites associated with glycerophospholids and sphingolipids metabolism form a complex network, which might partake in the pathogenesis of MP infection.

### Dysregulated lipid metabolism is associated with MPP disease severity

MP infection leads to membrane fusion damage and lipid metabolism changes. Whether there are differentially expressed metabolites associated with MPP disease severity is unclear. Therefore, this study also investigated if host-derived metabolites in circulation were implicated in MPP disease severity.

#### Metabolite profiles in severe MPPs vs. mild MPPs

Of the 91 MPP patients enrolled in this study, 54 patients had severe MPPs and 37 were mild cases. Compared with mild MPPs, severe cases were associated with greater pleural effusion, greater cardiac damage, liver damage, longer fever duration (*P* = 0.006), and longer hospital stay (*P* = 0.013). Indices of systemic inflammation, such as CRP (*P* = 0.004), also exhibited progressive increases as disease severity increased. Moreover, the level of laboratory parameters related to liver function (AST, ALT, TBA) and cardiovascular function (HBDB, LDH, CK-MB) were significantly higher in severe MPPs, compared with those in mild cases. In contrast, the levels of liver function indicator (ALB and ALP) and hematological markers (RBC, Plt, Hgb) were significantly higher in mild MPPs than those in severe subjects (Figure S4 and Table S6). Our observations are consistent with previous findings in laboratory-confirmed severe MPP cases where extrapulmonary complications indicative of serious disease were noted.

**Figure 7.**
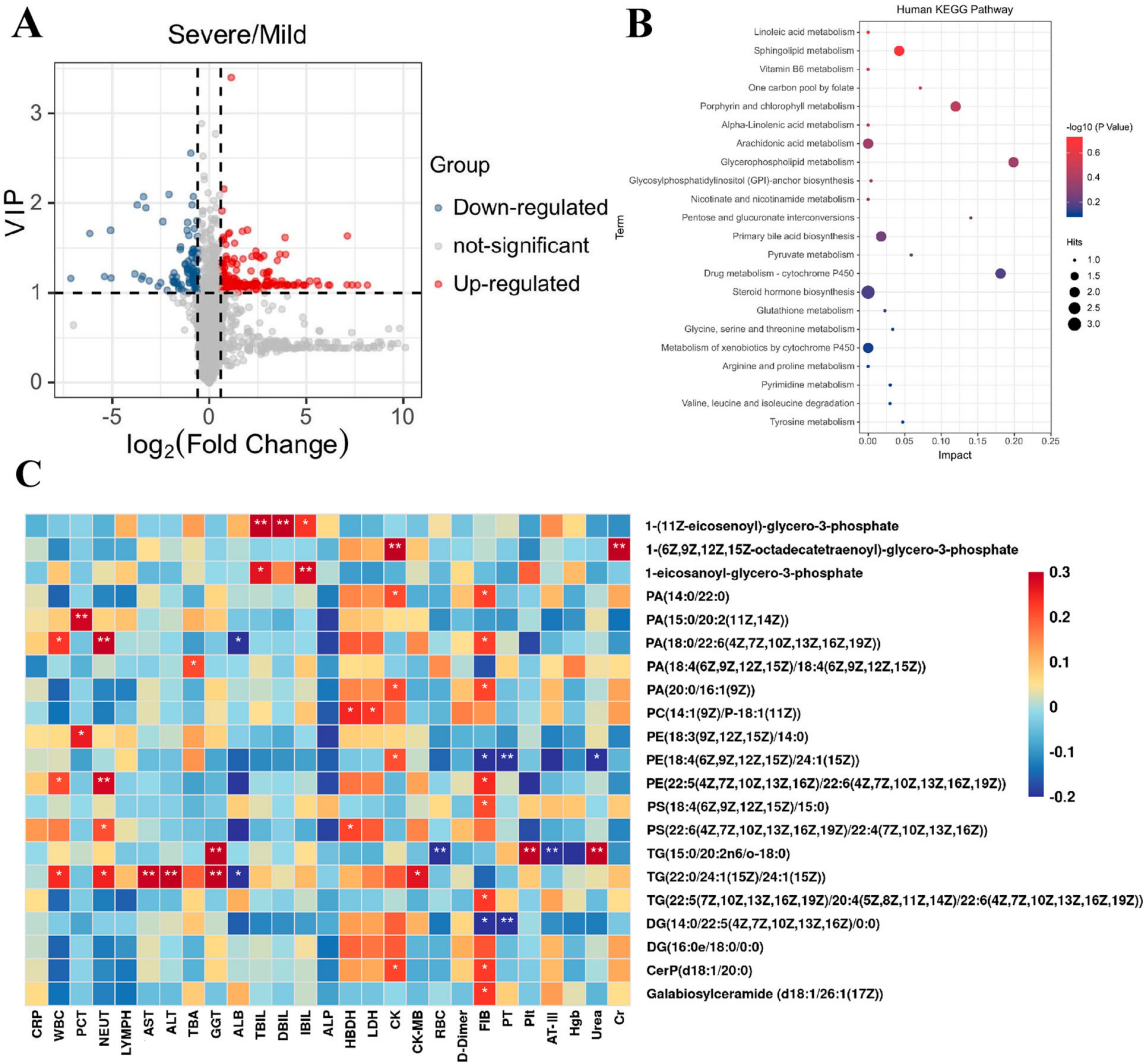
Dysregulated lipid metabolism is associated with disease severity in MPPs. (A) The volcano plot derived from a targeted metabolomic analysis illustrates the top serum metabolites that were increased (shown in red) or decreased (shown in blue) in severe MPP patients as compared with mild MPPs. (B) KEGG pathways that were significantly impacted in severe MPP disease. (C) Correlation analysis of differentially expressed metabolites and clinical indices between severe and mild MPPs. Red and blue represent positive and negative correlations, respectively. * means correlation *P-*value <0.05. ** means correlation *P*-value < 0.01.

To identify metabolites associated with severe disease, we further examined pathologically relevant metabolites in severe MPPs compared to mild subjects. A total of 247 metabolites were differently expressed between severe and mild MPPs (Table S7). Volcano plots from untargeted metabolomics analyses highlight the different metabolites that increased (red) or decreased (blue) in the sera of severe MPPs, as compared with mild subjects (Figure 7A). Interestingly, KEGG analysis also revealed a significant impact of severe disease on lipid metabolism, including glycerophospholipid and sphingolipid metabolism (Figure 7B).

#### Association between disease severity-related metabolites with relevant clinical indices

Next, we evaluated if differentially expressed metabolites in disease severity were correlated with relevant clinical indices. As shown in Figure 7(C), we observed that many differentially expressed metabolites displayed significant relationships with indices related to inflammation. For example, PA(18:0/22:6(4Z,7Z,10Z,13Z,16Z,19Z)) and PE(22:5(4Z,7Z,10Z,13Z,16Z)/22:6(4Z,7Z,10Z,13Z,16Z,19Z)), belonging to the class of glycerophospholipids, were associated with the number of WBCs. Moreover, some metabolites were associated with indices of extrapulmonary complications. For instance, we observed that the levels of triacylglycerol (TG) (22:0/24:1(15Z)/24:1(15Z)) was correlated with several liver function indicators including ALB, AST and ALT, which were also significantly different between severe MPPs and mild MPPs (Figure 7C). As reported previously, the liver function was assessed by lipid metabolism and inflammatory reaction induced by helicobacter pylori infection [30], supporting that the lipids metabolism involved in the process of liver damage. Additionally, the levels of PC(14:1(9Z)/P-18:1(11Z)) were positively correlated with the levels of HBDH and LDH, suggesting potential cardiovascular injury induced by MP infection (Figure 7C). The correlation between other lipid metabolites with clinical indices are shown in Table S8. Collectively, this suggests that lipid metabolites were correlated with extrapulmonary complications and may be involved in the disease severity of MPP.

## Discussion

Pathogen infection may alter the expression of proteins and metabolites [22, 31]. Traditionally, the proteins and metabolites circulate in the plasma through many different mechanisms such as secretion by humans after infection, stimulated production by antigen stimulation, or direct secretion by MP. Our previous study revealed several plasma proteins that were differentially expressed between MPP patients and controls [22]. According to the central dogma of molecular biology, DNA (genes) are transcribed to mRNA (transcripts) which are translated to proteins, and their activities result in the formation of small molecules (metabolites) [19]. As the downstream product of proteins, metabolites may be able to distinguish MPPs from controls, and directly clarify the pathogenesis of the MP infection.

Although metabolomics has been widely applied in some infectious and noninfectious diseases [11–18], little metabolomic information is available on MPP. LC/MS provides the most advanced technique for the selection of pathogen-derived metabolites with high stability and repeatability [32]. In this study, we used LC/MS metabolomics analysis to select MPP diagnostic biomarkers and investigate the metabolic profile of MPP. In clinical practice, MPPs are usually co-infected with other pathogens in children [33–35]. Thus HC and IDC are both used as controls in this study. After comparing MPPs with controls, 357 overlapping metabolites were identified to be associated with MPP.

Initially, it was difficult to select distinct metabolites to predict MPP because of the high-dimensional dataset. Thus we applied a random forest analysis to select the top metabolites that were differentially expressed between the three groups. Thirteen metabolites were identified among the 357 metabolites that had a high discriminatory value. Among them, 411.3208, 459.3493 and 568.5661 were identified and ROC analysis showed good sensitivity and specificity in the training cohort. In the independent testing cohort, when MPP patients were compared with HC or IDC, these three metabolites showed good results for AUC, sensitivity, and specificity.

The changes in the metabolome reflected changes in the biochemistry of host cells after MP infection. MP can incorporate certain lipids from their surroundings into its membrane and thereby influence lipid metabolism [36]. Cer (d18:0/18:0) belongs to the ceramide class, which is the main structural component of sphingolipids and plays a crucial part in activating cell signaling after pathogen infection [37]. Previous studies indicate that ceramides can cause alveolar and endothelial cell death, eventually leading to lung emphysema [38, 39]. Many sphingolipid species and their components such as ceramide, play important roles in regulating cellular signals such as growth, inflammation, and death, so it is not surprising that altered sphingolipid metabolism may be related to MP pathogenicity [40, 41]. In this study, we found that the level of plasma Cer (d18:0/18:0) was significantly changed in MP-infected patients compared with HCs and IDCs. Thus, elevated expression of sphingolipids in MPPs may be associated with the MP-host interaction during infection and further studies are required to elucidate its role.

LC/MS-based metabolomics is an untargeted method to select metabolic markers. The major advantage of using untargeted detection is that many unknown metabolites may also be discovered. In this study, we identified two new metabolites, 411.3208 (4a-formyl-5a-cholesta-8,24-dien-3b-ol) and 459.3493, which have potential diagnostic value in distinguishing MPPs from HCs or IDCs. 411.3208 (4a-formyl-5a-cholesta-8,24-dien-3b-ol) is closely associated with lipid transport and metabolism and the inflammatory response. Further functional analysis of 411.3208 (4a-formyl-5a-cholesta-8,24-dien-3b-ol) and 459.3493 were limited in this study and should be the focus of future analysis.

In addition, evaluation of alterations to metabolic pathways revealed that glycerophospholipid metabolism may be one of the main pathways involved in the pathogenesis of MP infection. Following MP infection, the lipid bilayer of cell membranes is susceptible to biomembrane fusion, and its structure involves the transcription of specific genes, cytoskeletal changes and changes in the nucleolus. Membrane fusion can also cause changes in receptor-identifying sites in the cell membrane, affecting signal delivery between cells and the production of cellular factors [29, 42]. Although lipid fusion plays an important role in MP infection, the specific effects of MP infection on lipid and downstream pathways remain unclear. In this study, we observed that glycerophospholipids, including LPA, LPC, PA, PC, PS and PE were significantly altered in plasma from MPP patients. The identification and implication of glycerophospholipid and sphingolipid metabolism pathways in MPP suggest membrane fusion damage induced by MP infection. Altered glycerophospholipid and sphingolipid metabolism pathways also suggest immune damage, which is another important factor in MP pathogenesis. Lipids are also involved in regulating a variety of biological processes such as immunity and inflammation [28]. As a novel class of inflammatory lipids, glycerophospholipids are also involved in several immune-mediated diseases, such as allergic airway disease and rheumatoid arthritis [43, 44]. This suggests that high expression of glycerophosholipds in plasma from MPPs not only indicates cellular membrane damage but also indicates the activation of immunity, consistent with several studies [29].

Changes in the metabolome are the ultimate response of a biological system to stimuli, and so, these changes may indirectly reflect pulmonary inflammation and lead to multiple severe extrapulmonary complications [19]. MP infection seriously affects host lipid metabolism and this might play an important role in the progression of MP disease and the occurrence of extrapulmonary complications. Here, we observed that several lipid metabolites, altered in mild and severe cases of MPP, were also significantly correlated with relevant clinical indices. In particular, we observed that TG were associated with several liver-damage-related indices. It has been reported that TG was accumulated in the liver during hepatomegaly [45], supporting our results. The effect of lipid metabolism on the development of severe MPP will be the focus of our future research.

It should be noted that this was a single-center prospective study with relatively small sample size and that missing values, which are common in LC–MS data, may affect data interpretation in studies with smaller sample sizes. Therefore, future large-sized cohort studies using higher sensitivity tests are warranted to confirm the findings in this study. The addition of asymptomatic MP patients will further reveal whether the metabolomics profile of asymptomatic patients can also be differentiated from mild/severe MPP as well as from HCs and IDCs.

In conclusion, differentially expressed metabolites in MPP plasma compared to HCs and IDCs were identified using metabolomics. Three biomarkers, 411.3208 (4a-formyl-5a-cholesta-8,24-dien-3b-ol), 459.3493 and 568.5661 (Cer (d18:0/18:0)) were independently verified to show good diagnostic value in distinguishing between MPPs and HCs or IDCs. A significant impact of MPP on lipid metabolism, especially pathways involving glycerophospholipid metabolism and sphingolipid metabolism, were identified and may contribute to the pathogenesis of MPP.

## Supplementary Material

Supplemental Material
