# Supplementary material for: Metabolomic analysis reveals potential biomarkers and the underlying pathogenesis involved in Mycoplasma pneumoniae pneumonia
Source: Emerg Microbes Infect. 2022 Feb 21;11(1):593–605. doi: 10.1080/22221751.2022.2036582 (PMC8865114; doi:10.1080/22221751.2022.2036582)
Supplement: Supplemental Material [file TEMI_A_2036582_SM6265.zip › Suppl files/Table S1.docx]

| **No.**^b^ | **Pathogen** |
| --- | --- |
| *1-3* | Parainfluenza virus |
| *4* | Parainfluenza virus *Haemophilus influenzae* |
| *5-7* | Respiratory syncytial virus |
| *8* | Respiratory syncytial virus, *Acinetobacter baumannii* |
| *9* | Respiratory syncytial virus, *Klebsiella pneumoniae* |
| *10-11* | Respiratory syncytial virus, *Streptococcus pneumoniae* |
| *12* | Respiratory syncytial virus, *Klebsiella pneumoniae* |
| *13-14* | Influenza A virus |
| *15* | Influenza A virus, Respiratory syncytial virus, |
| *16* | Influenza A virus, Respiratory syncytial virus, *Streptococcus pneumoniae* |
| *17-20* | Adenovirus |
| *21* | Influenza B virus |
| *22* | *Candida albicans* |
| *23-27* | *Streptococcus pneumoniae* |
| *28-29* | *Haemophilus influenzae* |
| *30-42* | Pathogen negative^c^ |

**Table S1. The causative agents of IDC^a^ patients**

a: infectious disease controls; b: Number of IDC patients; c: No pathogen was detected in pneumonia patients by culture and respiratory antigen detection
