# Supplementary material for: Metabolomic analysis reveals potential biomarkers and the underlying pathogenesis involved in Mycoplasma pneumoniae pneumonia
Source: Emerg Microbes Infect. 2022 Feb 21;11(1):593–605. doi: 10.1080/22221751.2022.2036582 (PMC8865114; doi:10.1080/22221751.2022.2036582)
Supplement: Supplemental Material [file TEMI_A_2036582_SM6265.zip › Suppl files/Table S2.docx]

Table S2 Details of the 13 metabolites with higher significance than the other metabolites.

|  |  |  |  | MPP vs IDC | | | MPP vs HC | | |
| --- | --- | --- | --- | --- | --- | --- | --- | --- | --- |
| m/z | time | HMDB | name | FC | P value | VIP | FC | P value | VIP |
| 225.1473 | 9.77 | HMDB0036583 | Methyl jasmonate | 0.344 | 1.08E-44 | 2.5689 | 0.40004 | 5.42E-46 | 2.049 |
| 229.9868 | 16.08 | HMDB0059911 | Paracetamol sulfate | 2.4725 | 1.17E-10 | 1.6598 | 0.36381 | 8.28E-22 | 1.7125 |
| 411.3208 | 9.71 | HMDB0001203 | 4a-Formyl-5a-cholesta-8,24-dien-3b-ol | 0.30524 | 3.16E-29 | 2.3676 | 0.16106 | 9.05E-61 | 2.1212 |
| 433.2303 | 17.7 | HMDB0007852 | LysoPA(0:0/18:2(9Z,12Z)) | 2.012 | 3.73E-10 | 1.6235 | 10.684 | 2.06E-27 | 1.8339 |
| 435.251 | 17.69 | HMDB0011133 | DHAP(18:0) | 4.2133 | 6.77E-16 | 1.9598 | 25.471 | 1.37E-25 | 1.7987 |
| 459.3493 | 13.26 | HMDB0032669 | (3beta,5alpha,9alpha,22E,24R)-3,5,9-Trihydroxy-23-methylergosta-7,22-dien-6-one | 3.5 | 1.33E-16 | 1.9916 | 2.4552 | 1.22E-15 | 1.5201 |
| 491.3396 | 12.55 | HMDB0033907 | Homodolicholide | 2.9916 | 5.93E-33 | 2.4333 | 2.75 | 4.85E-37 | 1.971 |
| 568.5661 | 15.38 | HMDB0011761 | Cer(d18:0/18:0) | 0.30984 | 2.88E-33 | 2.4382 | 0.27459 | 5.56E-46 | 2.049 |
| 763.5329 | 11.95 | HMDB0116652 | PG(a-13:0/i-22:0) | 2.4318 | 1.34E-37 | 2.4977 | 2.2106 | 1.03E-40 | 2.0074 |
| 901.4813 | 12.38 | HMDB0034063 | Asparasaponin II | 3.1698 | 1.77E-41 | 2.54 | 3.0721 | 5.92E-47 | 2.0555 |
| 941.4544 | 12.39 | HMDB0039291 | Medicoside H | 3.1624 | 2.42E-41 | 2.5387 | 3.098 | 1.07E-47 | 2.0603 |
| 963.474 | 12.38 | HMDB0009938 | PIP(16:0/22:5(7Z,10Z,13Z,16Z,19Z)) | 2.9463 | 5.08E-38 | 2.5027 | 2.8555 | 1.53E-43 | 2.0311 |
| 1004.368 | 13.41 | HMDB0001338 | Palmityl-CoA | 2.2041 | 4.78E-28 | 2.3437 | 2.0952 | 7.56E-31 | 1.8912 |

### MPP, *Mycoplasma pneumoniae* pneumonia; IDC, infectious disease control; HC, healthy control; FC, fold change; VIP: variable important in projection
