# Supplementary material for: Metabolomic analysis reveals potential biomarkers and the underlying pathogenesis involved in Mycoplasma pneumoniae pneumonia
Source: Emerg Microbes Infect. 2022 Feb 21;11(1):593–605. doi: 10.1080/22221751.2022.2036582 (PMC8865114; doi:10.1080/22221751.2022.2036582)
Supplement: Supplemental Material [file TEMI_A_2036582_SM6265.zip › Suppl files/Table S3.docx]

**Table S3. AUC, sensitivity, and specificity for ROC curves calculated at the optimal cut-off for the metabolites**

| **Different periods** | **Significant proteins by comparison groups** | **AUC** | **95%CI** | **Sensitivity** (%) | **Specificity** (%) |
| --- | --- | --- | --- | --- | --- |
| **Training cohort** | **MPP vs HC** | | | | |
|  | 411.3208 | 1 | 1-1 | 100 | 100 |
|  | 459.3493 | 1 | 1-1 | 100 | 100 |
|  | 568.5661 | 1 | 1-1 | 100 | 100 |
|  | **MPP vs IDC** | | | | |
|  | 411.3208 | 0.999 | 0.996-1 | 98.4 | 100 |
|  | 459.3493 | 1 | 1-1 | 100 | 100 |
|  | 568.5661 | 1 | 1-1 | 100 | 100 |
| **Testing cohort** | **MPP vs HC** | | | | |
|  | 411.3208 | 1 | 1-1 | 100 | 100 |
|  | 459.3493 | 0.884 | 0.779-0.989 | 85.7 | 85.7 |
|  | 568.5661 | 0.926 | 0.831-1 | 89.3 | 94.6 |
|  | **MPP vs IDC** | | | | |
|  | 411.3208 | 1 | 1-1 | 100 | 100 |
|  | 459.3493 | 0.864 | 0.762-0.967 | 71.4 | 100 |
|  | 568.5661 | 0.930 | 0.831 | 89.3 | 90 |

MPP, *Mycoplasma pneumoniae* pneumonia;

[AUC, area under the curve](https://blog.csdn.net/liweibin1994/article/details/79462554); ROC, receiver operating characteristic;

CI, confidence interval; IDC, infectious disease control;

HC, healthy control.
