# Supplementary material for: Metabolomic analysis reveals potential biomarkers and the underlying pathogenesis involved in Mycoplasma pneumoniae pneumonia
Source: Emerg Microbes Infect. 2022 Feb 21;11(1):593–605. doi: 10.1080/22221751.2022.2036582 (PMC8865114; doi:10.1080/22221751.2022.2036582)
Supplement: Supplemental Material [file TEMI_A_2036582_SM6265.zip › Suppl files/Table S5.docx]

**Table S5. Results of pathways calculated from enrichment analysis**

| KEGG Pathway | Total | Hits | P | Impact |
| --- | --- | --- | --- | --- |
| Glycerophospholipid metabolism | 36 | 5 | 0.007616 | 0.43397 |
| Sphingolipid metabolism | 21 | 3 | 0.035755 | 0 |
| Glycosylphosphatidylinositol (GPI)-anchor biosynthesis | 14 | 2 | 0.085656 | 0.00399 |
| Glycerolipid metabolism | 16 | 2 | 0.10802 | 0.02648 |
| Retinol metabolism | 17 | 2 | 0.11972 | 0.24227 |
| Ether lipid metabolism | 20 | 2 | 0.15652 | 0 |
| Linoleic acid metabolism | 5 | 1 | 0.16547 | 0 |
| Biosynthesis of unsaturated fatty acids | 36 | 2 | 0.36795 | 0.13636 |
| alpha-Linolenic acid metabolism | 13 | 1 | 0.37596 | 0 |
| Fatty acid elongation | 39 | 2 | 0.40655 | 0.06363 |
| Fatty acid degradation | 39 | 2 | 0.40655 | 0.10496 |
| Citrate cycle (TCA cycle) | 20 | 1 | 0.5167 | 0.06592 |
| Pyruvate metabolism | 22 | 1 | 0.55084 | 0.17937 |
| Glycolysis / Gluconeogenesis | 26 | 1 | 0.61215 | 0.08802 |
| Phosphatidylinositol signaling system | 28 | 1 | 0.63965 | 0.00152 |
| Arachidonic acid metabolism | 36 | 1 | 0.73175 | 0 |
| Pyrimidine metabolism | 39 | 1 | 0.75995 | 0.0068 |
| Tryptophan metabolism | 41 | 1 | 0.77711 | 0 |
| Steroid biosynthesis | 42 | 1 | 0.78524 | 0 |
| Steroid hormone biosynthesis | 85 | 2 | 0.81681 | 0.02177 |
| Fatty acid biosynthesis | 47 | 1 | 0.82169 | 0 |
| Purine metabolism | 65 | 1 | 0.90921 | 0.00234 |

“Total” is the total number of compounds in the pathway; “Hits” is the matched number from the user uploaded data; “P” is the original P value that was calculated from the enrichment analysis; “Impact” is the pathway impact value that was calculated from pathway topology analysis
