# Supplementary material for: Metabolomic analysis reveals potential biomarkers and the underlying pathogenesis involved in Mycoplasma pneumoniae pneumonia
Source: Emerg Microbes Infect. 2022 Feb 21;11(1):593–605. doi: 10.1080/22221751.2022.2036582 (PMC8865114; doi:10.1080/22221751.2022.2036582)
Supplement: Supplemental Material [file TEMI_A_2036582_SM6265.zip › Suppl files/Table S6.docx]

Table S6: Demographic and clinical characteristics among children hospitalized for MPP

| Respiratory manifestations and inflammatory markers | Total (n=91) | Sub-group | | |
| --- | --- | --- | --- | --- |
|  |  | Mild MPP (n =37) | Severe MPP (n =54) | P value ^a^ |
| Gender (male/female) | 51/40 | 17/20 | 34/20 | 0.095 |
| Age (years) ^b^ | 6.9±8.2 | 7.1±2.8 | 6.6±2.3 | 0.145 |
| Days of hospitalization ^b^ | 10.0±4.3 | 8.8±4.4 | 10.8±4.0 | 0.013 |
| Fever duration (Day) ^b^ | 2.6 ±1.9 | 2.0±1.7 | 3.0±2.0 | 0.006 |
| Extrapulmonary complications, n (%) | | | | |
| Cardiac damage | 18(19.8) | 0(0) | 18(33.3) | 0.000 |
| Liver damage | 10(11.0) | 0(0) | 10(18.5) | 0.000 |
| Pleural effusion | 26(28.6) | 0(0) | 26(48.1) | 0.000 |
| Inflammation markers ^b^ | | | | |
| CRP, mg/L | 31.0±36.5 | 18.9±14.7 | 39.4±44.2 | 0.004 |
| PCT, ng/ml | 0.4±0.9 | 0.6±1.5 | 0.3±0.08 | 0.099 |
| WBC, ×10^9^/L | 7.9±3.3 | 7.4±2.2 | 8.3±3.8 | 0.113 |
| NEUT, ×10^9^/L | 5.2±2.7 | 4.8±1.9 | 5.5±3.1 | 0.130 |
| LYMPH, ×10^9^/L | 1.9±1.0 | 1.9±0.6 | 2.0±1.1 | 0.292 |
| Liver function indicators ^b^ | | | | |
| AST, umol/L | 43.0±67.9 | 26.9±8.6 | 54.0±86.5 | 0.007 |
| ALT, umol/L | 29.5±63.1 | 16.1±6.8 | 38.6±80.8 | 0.047 |
| TBA, umol/L | 6.1±4.7 | 5.0±3.7 | 6.8±5.2 | 0.043 |
| GGT, umol/L | 17.2±17.1 | 15.8±14.6 | 18.2±18.6 | 0.260 |
| ALB, umol/L | 36.0±3.9 | 37.7±2.9 | 34.9±4.2 | 0.000 |
| ALP, umol/L | 128.3±40.5 | 146.6±42.0 | 115.8±34.6 | 0.000 |
| TBIL, umol/L | 7.8±2.9 | 8.0±3.4 | 7.7±2.5 | 0.301 |
| DBIL, umol/L | 1.3±1.6 | 1.5±2.4 | 1.2±0.6 | 0.202 |
| IBIL, umol/L | 6.5±1.8 | 6.5±1.5 | 6.5±2.1 | 0.463 |
| Cardiovascular markers ^b^ | | | | |
| HBDH, umol/L | 296.0±140.3 | 243.5±58.8 | 331.9±166.8 | 0.001 |
| LDH, umol/L | 394.0±226.9 | 306.9±81.8 | 453.7±272.0 | 0.001 |
| CK, U/L | 120.8±164.0 | 104.3±146.2 | 132.1±175.7 | 0.215 |
| CK-MB, umol/L | 14.0±6.1 | 12.6±3.3 | 15.0±7.4 | 0.031 |
| Hematologic markers ^b^ | | | | |
| RBC, ×10^9^/L | 4.4±0.4 | 4.6±0.4 | 4.3±0.4 | 0.001 |
| D-Dimer, mg/L | 0.9±1.7 | 0.4±0.3 | 1.3±2.1 | 0.008 |
| FIB, g/L | 4.2±0.9 | 4.1±0.5 | 4.3±1.1 | 0.124 |
| PT, min | 12.3±1.6 | 12.3±1.0 | 12.1±1.9 | 0.401 |
| Plt, 10^9^/L | 319.6±100.2 | 340.8±93.4 | 305.1±102.9 | 0.048 |
| AT-Ⅲ, % | 111.5±23.9 | 112.7±27.8 | 110.6±21.1 | 0.237 |
| Hgb, g/L | 124.0±10.4 | 127.4±8.2 | 121.7±11.2 | 0.005 |
| Urinary markers ^b^ | | | | |
| Urea, nmol/L | 3.3±1.1 | 3.4±1.3 | 3.2±0.9 | 0.166 |
| Cr, umol/L | 39.3±8.6 | 40.3±8.9 | 38.6±8.5 | 0.179 |

CRP, C-reactive protein; WBC, white blood cell; PCT, procalcitonin; NEUT%, neutrophil%; LYMPH%, [lymphocyte](https://fanyi.so.com/?src=onebox#lymphocyte)%; AST, aspartate aminotransferase; ALT, alanine aminotransferase; TBA, total bileacide; GGT, γ-glutamyl transpeptidase; ALB, albumin; TBIL, total bilirubin; DBIL, direct bilirubin; IBIL; indirect bilirubin; ALP, alkaline phosphatase; HBDB, hydroxybutyrate dehydrogenase; LDH, lactic dehydrogenase; CK, creatine kinase; CK-MB, phosphocreatine kinase isoenzyme; RBC, red blood cell; FIB, fibrinogen; PT, total protein; PLT, platelet; Hgb, hemoglobin; Cr, urinary creatinine; ^a^ P value between severe and mild MPPs; ^b^ Data are presented as the mean ± SD.
