# Supplementary figures and images for: Metabolomic analysis reveals potential biomarkers and the underlying pathogenesis involved in Mycoplasma pneumoniae pneumonia
Source: Emerg Microbes Infect. 2022 Feb 21;11(1):593–605. doi: 10.1080/22221751.2022.2036582 (PMC8865114; doi:10.1080/22221751.2022.2036582)

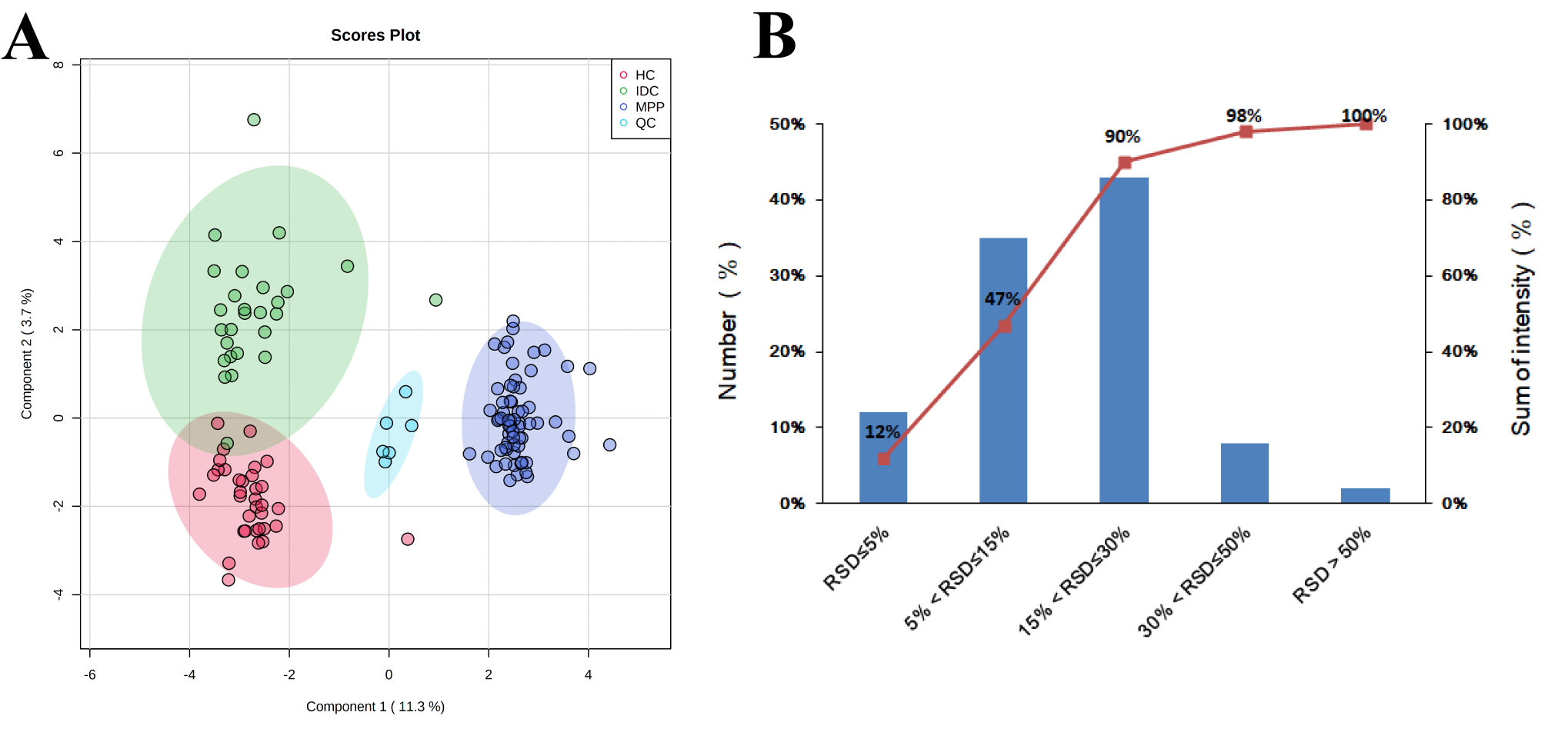

Supplement: Supplemental Material [file TEMI_A_2036582_SM6265.zip › Suppl files/Figure S1.jpg]

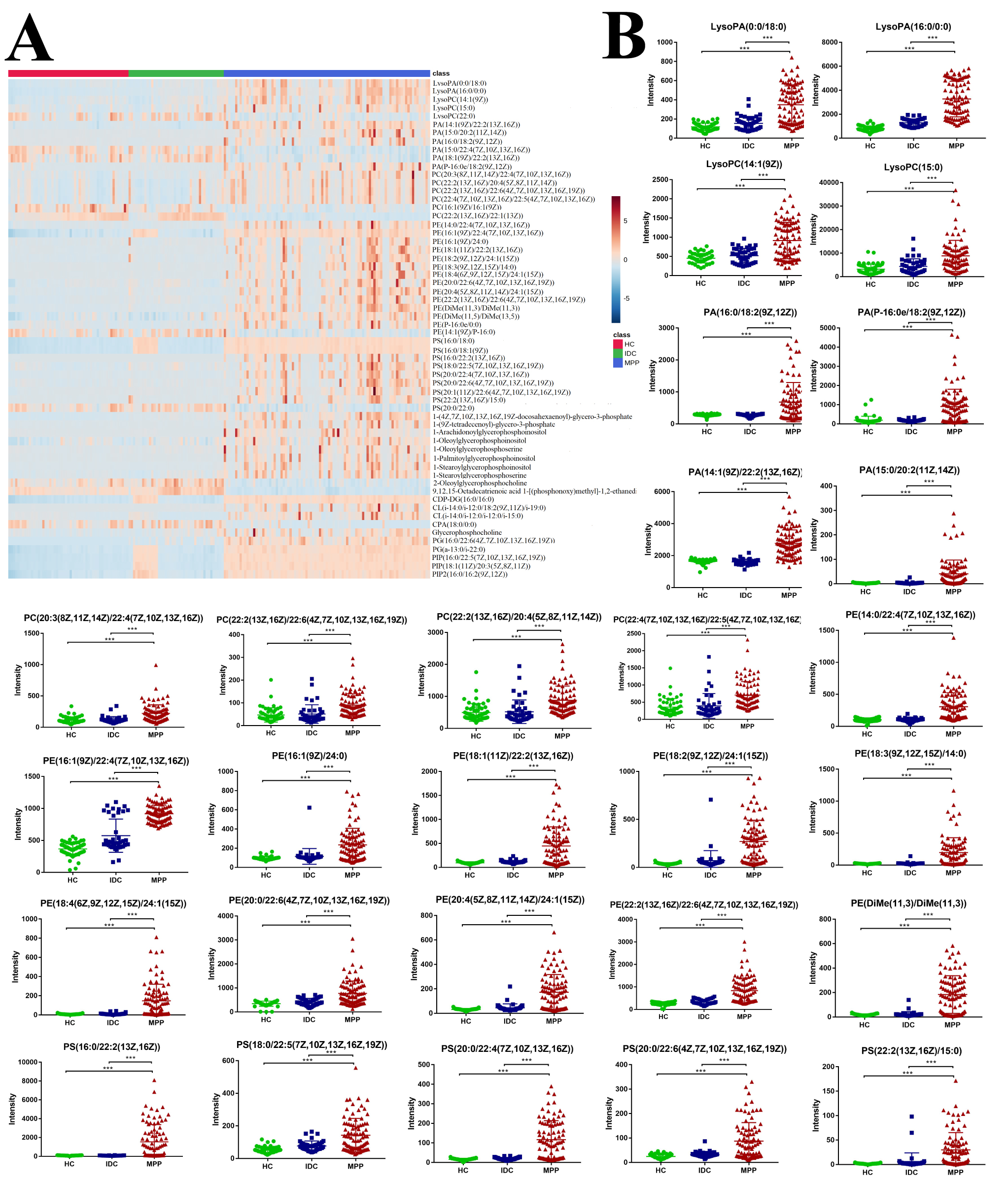

Supplement: Supplemental Material [file TEMI_A_2036582_SM6265.zip › Suppl files/Figure S2.jpg]

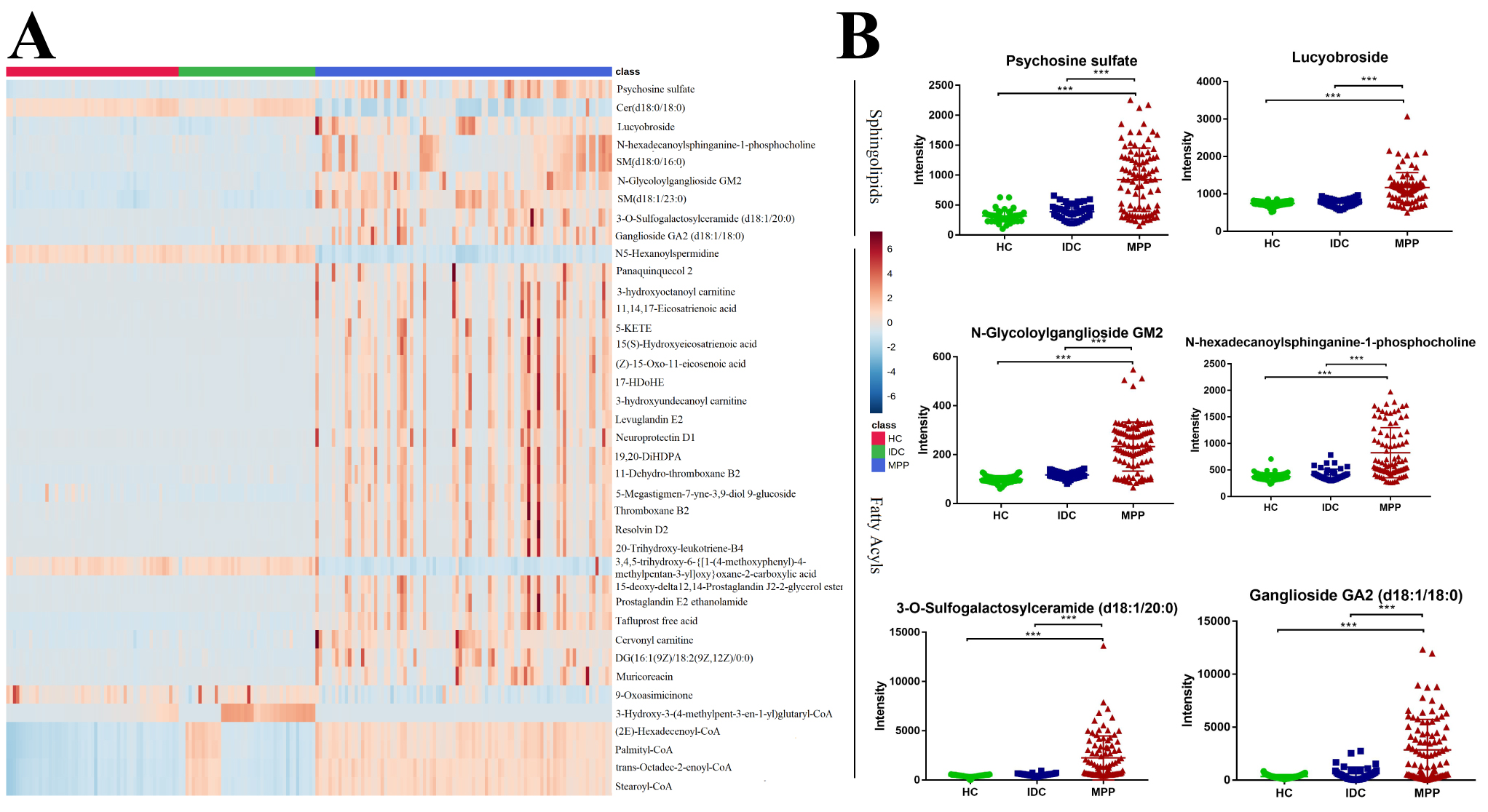

Supplement: Supplemental Material [file TEMI_A_2036582_SM6265.zip › Suppl files/Figure S3.jpg]

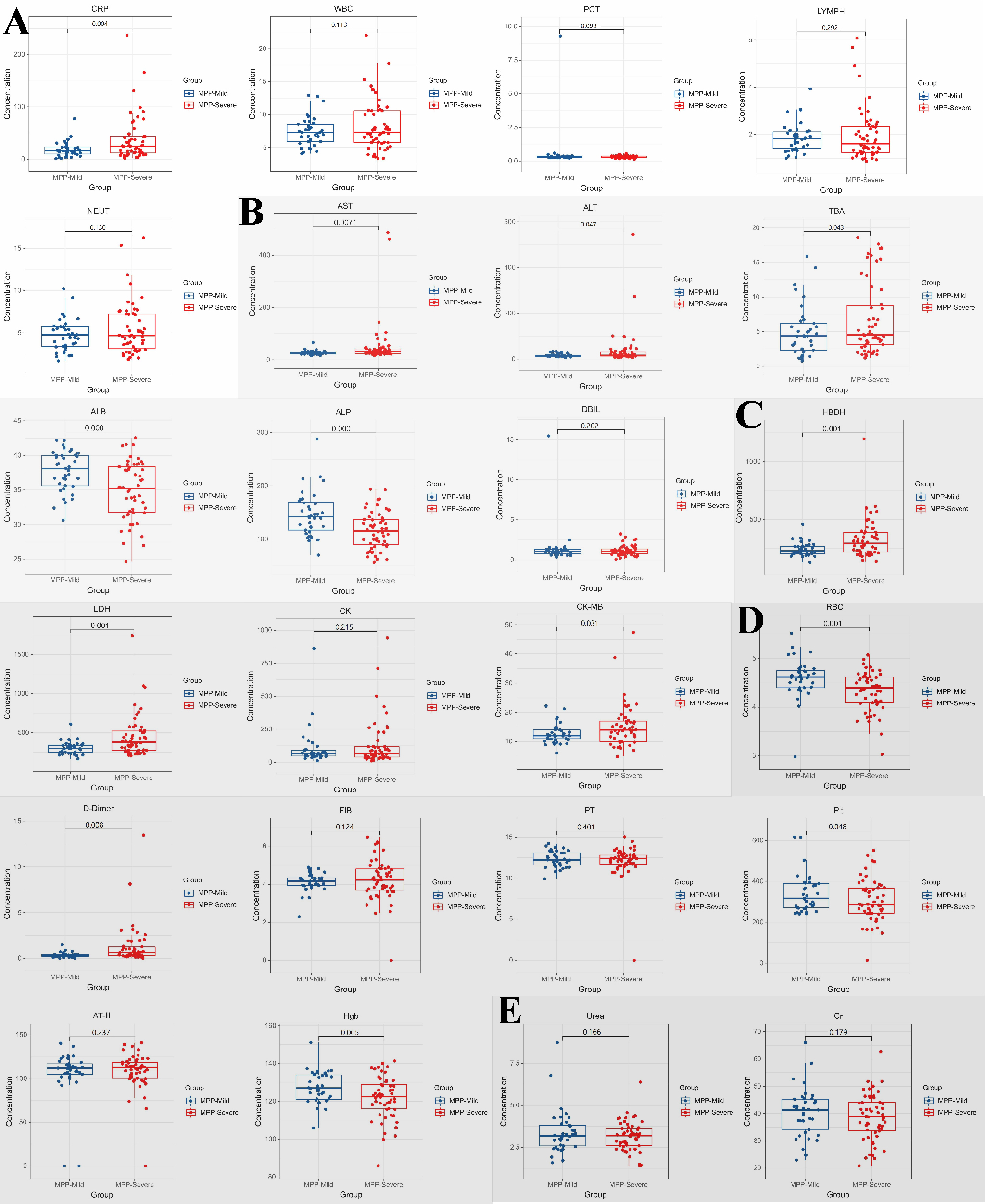

Supplement: Supplemental Material [file TEMI_A_2036582_SM6265.zip › Suppl files/Figure S4.jpg]
