# Supplementary material for: Metabolomic analysis reveals potential biomarkers and the underlying pathogenesis involved in Mycoplasma pneumoniae pneumonia
Source: Emerg Microbes Infect. 2022 Feb 21;11(1):593–605. doi: 10.1080/22221751.2022.2036582 (PMC8865114; doi:10.1080/22221751.2022.2036582)
Supplement: Supplemental Material [file TEMI_A_2036582_SM6265.zip › Suppl files/_2_Mauscript-1_Supplementary_LL_clean copy.doc]

**Supplementary**

**Metabolomics Analysis Reveals Potential Biomarkers and the Underlying Pathogenesis Involved in *Mycoplasma Pneumoniae* Pneumonia**

Jieqiong Li ^1,*^, Laurence Don Wai Luu ^2,*^, Xiaoxia Wang ^3^, XiaoDai Cui ^4^, Xiaolan Huang ^4^, Jin Fu ^4^, Xiong Zhu ^3, †^, Zhenjun Li ^5, †^, Yi Wang ^4, †^ and Jun Tai ^6, †^

^1^ Department of Respiratory Disease, Beijing Pediatric Research Institute, Beijing Children’s Hospital, Capital Medical University, National Center for Children’s Health, Beijing 10045, P. R. China.

^2^ School of Biotechnology and Biomolecular Sciences, University of New South Wales, Sydney, Australia.

^3^ Central & Clinical Laboratory of Sanya People’s Hospital, Sanya, Hainan 572000, P. R. China.

^4^ Experimental Research Center, Capital Institute of Pediatrics, Beijing 100020, P. R. China.

^5^ State Key Laboratory for Infectious Disease Prevention and Control, National Institute for Communicable Disease Control and Prevention, Chinese Center for Disease Control and Prevention, Beijing 102200, P.R. China

^6^ Department of Otolaryngology, Head and Neck Surgery, Children's Hospital Capital Institute of Pediatrics, Beijing 100020, P. R. China.

**Corresponding author:**

Xiong Zhu; *E-mail*: zhuxiong6@163.com

Zhenjun Li; *E-mail*: lizhenjun@icdc.cn

Yi Wang; *E-mail*: [wildwolf0101@163.com](mailto:wildwolf0101@163.com) (Handing the correspondence);

Jun Tai; *E-mail*: trenttj@163.com;

**Figure S1. Metabolomic Data Quality control.**

(A) Reliability investigation of the analytical method using QC samples (n = 6). Score plot of Orthogonal Partial Least Squares Discrimination Analysis (OPLS-DA) (HC, healthy control; IDC, infectious disease control; MPP, *mycoplasma pneumoniae* pneumonia; QC, quality control), (B) Relative standard deviation (RSD)% of all detected variables. Number (%), percentage of number of variables. Sum of intensity (%), percentage of total response of variables.

**Figure S2. Expression of Glycerophospholipids in Each Group.**

(A) Hierarchical clustering analysis revealed major classes of plasma glycerophospholipids including glysophosphatidic acid (LysoPA), lysophosphatidylcholine (LysoPC), phosphatidic acid (PA), phosphatidylcholine (PC), Glycerophosphoserines (PS) and phosphatidylethanolamines (PE) were significantly increased in MPPs compared to HCs and IDCs. (B) Significant changes were seen in the levels of some glycerophospholipids in the plasma of MPP samples.

**Figure S3. Expression of Sphingolipids in Each Group.**

(A) Hierarchical clustering analysis revealed most sphingolipids and fatty acyls were significantly increased in MPPs compared to controls. (B) Significant changes were seen in the levels of sphingolipids in the plasma of MPP samples.

**Figure S4. Comparison of Clinical indices between Mild and Severe MPPs.**

(A) Expression of inflammation markers. (B) Expression of liver function indicators. (C) Expression of cardiovascular markers. (D) Expression of hematologic markers. (E) Expression of urinary markers.
